# Multi-layer 3D chirality: its enantioselective synthesis and aggregation-induced emission

**DOI:** 10.1093/nsr/nwaa205

**Published:** 2020-04-02

**Authors:** Junliang Zhang, László Kürti

**Affiliations:** Department of Chemistry, Fudan University, China; Department of Chemistry, Rice University, USA

Chirality has been extensively studied in interdisciplinary fields for almost two centuries since its ubiquity was discovered by Louis Pasteur in 1848, when he successfully separated a pair of enantiomers. Indeed, chirality is present at all levels in nature—in the form of subatomic particles, macromolecules such as proteins and DNA, microscopic living organisms such as helical bacteria and even in the form of macroscopic objects such as sea shells and spiral galaxies. It is undeniable that the concept of chirality and the field of stereochemistry, which studies the three-dimensional (3D) structure and relationships of molecules, has had a monumental impact on chemical, biomedical and material sciences [1]. Not surprisingly, the design and synthesis of an ever-increasing number of small-molecule pharmaceuticals as well as optical materials heavily depend on our deep understanding and exploitation of molecular chirality. In chemistry, chirality is divided into point/central, spiro, axial, helical/planar as well as the multi-layer versions of helical/planar chirality [2]. With the exception of multilayer chirality all the other types exist widely in nature.

Professor Guigen Li's teams at Nanjing University and Texas Tech University have recently discovered and characterized the first examples of multi-layer 3D chirality where the layers are not bridged together; this is a novel form of chirality which is different from traditional planar and helical chirality (i.e. a highly compacted chiral fold held together primarily by π-stacking interactions), and the enantioselective synthesis of this framework has been achieved [3]. This new chiral framework, a multi-layer organic framework (M-LOF), has unique *C_2_*- and/or pseudo *C_2_*-symmetry and features three layers that are arranged in a nearly parallel fashion: a top, a middle and a bottom aromatic ring. Interestingly, this multi-layer type framework displays elements of both planar and axial chirality (i.e. rotational stereoisomerism)—in the compounds that exhibit multi-layer 3D chirality the top and the bottom layers have restricted rotation relative to each other. This means, in essence, that if either the top or the bottom layer is removed, multi-layer 3D chirality would no longer exist due to free rotation.

**Scheme 1.**
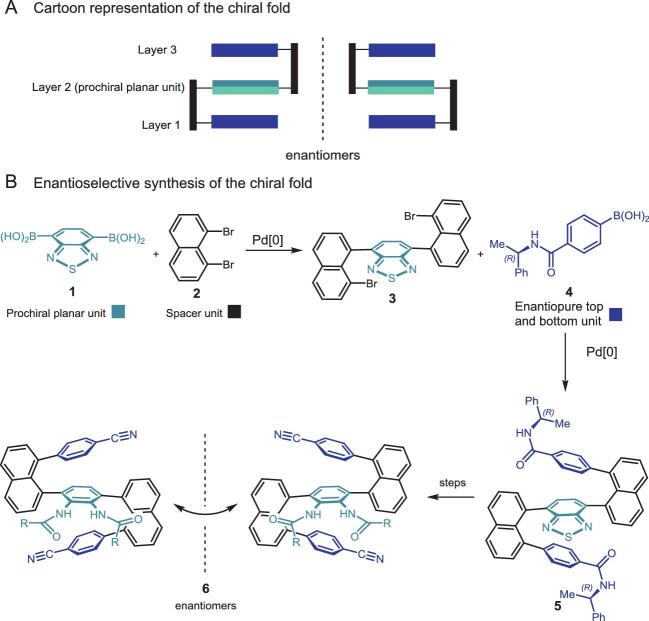
Asymmetric synthesis of compounds exhibiting multi-layer 3D chirality.

Professor Li's group isolated the first multi-layer 3D chiral molecule during their ongoing project on GAP (Group-Assisted-Purification) chemistry that takes advantage of certain functional groups that allow the greener and more atom economical synthesis of chiral building blocks by avoiding the formation of non-crystalline intermediates [4]. The synthesis of compounds that exhibit C–N bond-based multi-layer 3D chirality of *pseudo C_2_*-symmetry was achieved via employing a double Buchwald-Hartwig cross-coupling, while compounds that exhibit the C–C bond-based multi-layer 3D chirality of *C_2_*-symmetry were obtained via a double Suzuki-Miyaura coupling and several additional steps. Enantiomers of the former were obtained via preparative chiral HPLC, and those of the latter through asymmetric synthesis (Scheme 1).

**Figure 1.**
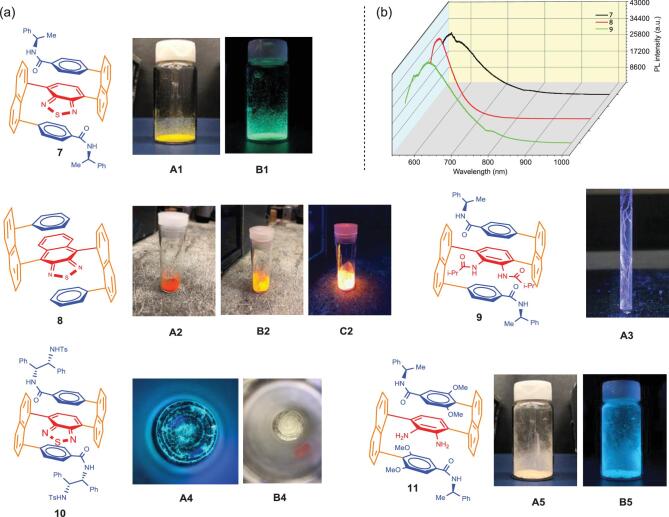
(a) Luminescence of samples under UV light (365 nm): **A1** and **A5** without UV irradiation, **B1** and **B5** with UV irradiation; fluorescence images of **8** under different physical conditions (**A2**, appearance under natural light; **B2**, UV irradiation in natural background; **C2**, UV irradiation in dark background); spiro textile-type of macro-chirality of **9** formed inside (**A3**) NMR tube under UV irradiation; macro-chirality phenomenon of **10**: (**A4**) image under UV light (365 nm), (**B4**) image under natural light after rotavapor evaporation. (b) Photoluminescence (PL) spectra of **7**, **8** and **9** as solid test samples; excitation wavelength (λ_ex_): 532 nm.

The synthetic sequence en route to compounds that exhibit C–C bond-based multi-layer 3D chirality utilized benzo[c][1,2,5]thiadiazole-4,7-diyldiboronic acid (**1**) as a key building block. This compound was used as the central bifunctional coupling partner (i.e. which ultimately serves as the bridging middle aromatic ring of the final multi-layer structure) in the subsequent double Suzuki-Miyaura cross-coupling to furnish compound **3**. The chirality was controlled by using two identical chiral amide scaffolds, derived from (*R*)-methylbenzylamine (**4**), which were attached to the naphthalene rings via two Suzuki-Miyaura cross-coupling reactions. Finally, the thiadiazole rings of multi-layer 3D enantiomers were opened to give the corresponding diamino products (**6**) that were isolated in the N-protected form. Scheme 1 shows the structure of a pair of enantiomers side-by-side. Macro-chirality of some chiral 3D molecules can be directly observed with the naked eye without the need for instrumentation (Fig. 1(a)A3 and A4). Many of the 3D products showed strong fluorescence sensitivity even in their solid forms under UV light at 365 nm (Fig. 1(a)) and displayed aggregation-induced emission (AIE) [5] properties (Fig. 1(b)). Furthermore, the enantiomers of several multi-layer 3D chiral compounds showed unusually high optical rotational data. In summary, it is anticipated that this work will have a broad impact on chemical, medicinal, biomedical as well as material sciences including research that focuses on synthesis and exploitation of optoelectronic materials.

***Conflict of interest statement.*** None declared.
